# Assessment of the Effects of Cigarette Smoke on the Renal Morphology of WISTAR Rats

**DOI:** 10.1002/jat.4872

**Published:** 2025-08-02

**Authors:** Cristiane Gomes Fernandes Freire, Luana Aparecida Cunha, Mateus Andrade Stecca, Isabele Masson de Brito, Rafaella Riccio da Silva, Thiago Donizeth da Silva, Margarida Pereira Santos, Márcia Cristina Bizinotto de Assunção, Geraldo José Medeiros Fernandes, Evelise Aline Soares

**Affiliations:** ^1^ Faculty of Medicine Federal University of Alfenas Alfenas Minas Gerais Brazil; ^2^ Faculty of Dentistry Edson Antônio Vellano University (UNIFENAS) Alfenas Minas Gerais Brazil; ^3^ Postgraduate Program in Biological Sciences Federal University of Alfenas Alfenas Minas Gerais Brazil; ^4^ Postgraduate Program in Nursing Federal University of Alfenas Alfenas Minas Gerais Brazil; ^5^ Institute of Biomedical Sciences Federal University of Alfenas Alfenas Minas Gerais Brazil

**Keywords:** cigarette smoke, kidney, morphology, smoking

## Abstract

Smoking is a major public health concern worldwide. The toxic components of cigarettes, such as nicotine, can cause abuse, dependence, and tissue damage. This study aimed to evaluate the effects of exposure to cigarette smoke on the renal morphology of Wistar rats. For this purpose, 12 male rats were used, divided into two groups (*n* = 6): CT Group (control) and CG Group (cigarette). The CG Group was exposed to smoke from six cigarettes per day. After 12 weeks, the rats were euthanized, and the kidneys were collected, weighed, and processed histologically for renal tissue analysis. Although the increase in renal weight was not statistically significant (*p* = 0.479), the CG Group exhibited significantly reduced values for glomerular diameter, capsular area (*p* < 0.0001), subcapsular area (*p* = 0.0045), and glomerular tuft area (*p* = 0.0049) compared to the CT Group. These results indicate that cigarette smoke exposure induces morphological changes in renal structure, even in the absence of significant weight alteration.

## Abstract 2

This study evaluated the effects of cigarette smoke exposure on the renal morphology of Wistar rats. After 12 weeks, significant reductions in glomerular diameter, capsular area, subcapsular area, and glomerular tuft area were observed in the exposed group compared to controls. Although kidney weight was not significantly affected, these findings demonstrate that cigarette smoke causes notable histological changes in renal structure, highlighting its potential for tissue damage even without gross anatomical alterations.

## 1 Introduction

Smoking is recognized globally as a major public health concern, associated with numerous diseases including cancer, respiratory, and cardiovascular disorders. In Brazil, one estimates that approximately 157,000 people die prematurely each year due to diseases caused by this condition. In this context, smokers are two to three times more likely to develop kidney cancer compared to non‐smokers, due to less physical resistance and greater ease in becoming ill, demonstrating that tobacco exposure also affects renal health, increasing the risk for kidney cancer and chronic kidney disease (CKD). Carcinogens and toxins in cigarette smoke are absorbed into the bloodstream and reach the kidneys in high concentrations, leading to substantial exposure of renal cells.

Despite being a risk factor considered modifiable, smoking directly affects the urinary system, made up of a pair of kidneys and ureters, a urinary bladder, and a urethra. The kidneys, specifically, are retroperitoneal organs, bean‐shaped, surrounded by a capsule of connective tissue, with a medial concave margin where the renal hila are centered, through which the renal arteries enter and the renal veins and ureters exit (Moore et al. 2024). Regarding histology, it is possible to internally identify two macroscopic regions: the cortex and the medulla (Junqueira et al. 2017).

The cortical region, right below the capsule, houses the renal corpuscles (glomerulus and Bowman's capsule), as well as proximal and distal convoluted tubules. In the medullary region, there are the loops of Henle. These structures, together, form the morphofunctional unit of the kidneys, the nephron. Each distal convoluted tubule opens into the collecting ducts, which empty the urine into the renal pelvis through the projection of the papilla into smaller calyces, which unite into 2–4 larger calyces. The kidney has several functions related to the body's homeostasis, the most important of which is the maintenance of water and electrolyte balance (Silverthorn 2017). Through plasma filtration, the kidneys allow the excretion, secretion, and reabsorption of substances, such as water and ions, that occur according to the body's needs. Therefore, this organ influences the regulation of blood pressure, osmolality, and blood electrolyte concentration (Jaimes, Zhou, et al. 2021).

The kidneys play a fundamental role in regulating blood pressure in the long term, excreting variable amounts of sodium and water, and in the short term, secreting hormones and substances such as renin, which contribute to the formation of vasoactive products such as angiotensin II (Guyton and Hall 2020). Furthermore, the functionality of the renal system, according to Silverthorn (2017), is physiologically integrated with behaviors such as thirst and urination.

Due to their ability to excrete and reabsorb bicarbonate ions (HCO_3_) and protons (H^+^), the kidneys actively participate in the regulation of plasma pH. Although not as fast as pulmonary action, renal participation in this acid–base balance is crucial, as the pH of the extracellular fluid must be maintained in a range that allows little variation. When plasma acidification occurs, the kidneys excrete H^+^ and conserve bicarbonate ions. Likewise, when blood pH increases, the kidneys reabsorb H^+^ and excrete bicarbonate ions. Additionally, the kidneys are the only way to eliminate some acids from the body, such as sulfuric and phosphoric acids. Furthermore, the hormonal production of the kidneys is not limited to the release of the aforementioned renin, as they are also responsible for almost all the production and secretion of circulating erythropoietin, a hormone that stimulates the production of red blood cells by acting on hematopoietic stem cells in the bone marrow (Gueutin et al. 2012). According to the authors, the renal preponderance of erythropoietin production is such that patients with severe renal dysfunction or whose kidneys have been removed develop severe anemia due to the reduction in the production and secretion of this important hormone. According to Kurtz (2017), there is a third endocrine role of the kidneys: the conversion of vitamin D3 into an active hormone that regulates Ca_2_^+^ balance.

Firstly, when we refer to smoking, it must be defined as a chronic disease caused by dependence on the nicotine present in tobacco products. In this context, it is essential to understand the mechanisms of action of tobacco on body tissues because chronic use of this substance is associated with deaths from cancer and is therefore a public health problem.

Concerning these perspectives, it appears that smoking is a risk factor for CKD (Elihimas Júnior et al. 2014). Furthermore, it is demonstrated that the rate of urinary excretion of elevated albumin and microalbuminuria is higher in smokers compared to non‐smoking individuals in the general population (Orth 2002). According to data from the Multiple Risk Factor Intervention Trial (MRFIT), smoking increases the risk of end‐stage renal failure in men. Smoking is particularly “nephrotoxic” in older individuals, those with systemic arterial hypertension, and in people who already have some degree of kidney disease (Lee and Fariss 2017).

A study carried out by Jiang et al. (2019) demonstrated that cigarette smoke did not affect the levels of plasma glucose, hemoglobin A1c, high‐density lipoprotein cholesterol, low‐density lipoprotein cholesterol, or non‐esterified fatty acids in control, and diabetic rats under experimental conditions. However, it significantly increased diabetes‐induced glomerular hypertrophy and excretion of urinary kidney injury molecule‐1 (KIM‐1) and neutrophil gelatinase‐associated lipocalin (NGAL), suggesting exacerbation of diabetic kidney injury. In this experimental mode, cigarette smoke promoted macrophage infiltration and fibrosis in the diabetic kidney. As expected, cigarette smoke increased oxidative stress in both control and diabetic rats.

Given the above, considering the large number of individuals who smoke, the importance of the topic, and the scarcity of studies referring to renal histomorphometric changes due to exposure to cigarette smoke, the present study aimed to evaluate the effects of smoking on the renal morphology of Wistar rats under experimental mode.

## 2 Material and Methods

### 2.1 Ethical Principles

This study was conducted in accordance with the Brazilian guidelines for the care and use of laboratory animals, as outlined in the Arouca Law (Law No. 11,794/2008) and in the Normative Resolution CONCEA No. 57, dated December 6, 2022, which specifically addresses the breeding, maintenance, and experimentation involving rodents and lagomorphs. The project was duly reviewed and approved by the Animal Ethics Committee (CEUA) of the José do Rosário Vellano University (UNIFENAS), protocol no. 02A/2008.

### 2.2 Animal Protocol

Twelve male Wistar albino rats (40 days old, 180 ± 25 g) were provided and maintained in the vivarium of the postgraduate program at José do Rosário Vellano University, with temperature control and a 12‐h light/dark cycle. The animals were kept in conventional cages with the floor lined with wood shavings. Throughout the experiment, the animals had ad libitum access to food and water, receiving the same solid diet (Nuvilab CR‐1 Autoclavable food). The rats were divided into two random groups (*n* = 6), the Control Group (CT) and the Cigarette Group (CG). In the CT Group (Control) all animals received Nuvilab CR‐1 Autoclavable rodent feed as solid diet and water ad libitum as liquid diet. In the CG Group (cigarette) the animals were exposed to smoke from six cigarettes with a concentration of 1.3 mg of nicotine, 16.5 mg of tar, and 15.2 mg of carbon monoxide over 12 weeks. This contact occurred daily in the morning, with an average daily exposure time of 15–20 min. Initially, the animals went through an adaptation period of 6 days, with gradual exposure to cigarette smoke, starting with one cigarette on the first day and increasing to six cigarettes on the sixth day (César‐Neto et al. 2006; Bueno et al. 2011). For exposure to smoke, the rats were placed in a transparent acrylic box measuring 45 × 25 × 20 cm^3^, containing a chamber with ventilation holes and other smaller holes in the upper part, in which cigarettes were placed for the propagation of smoke (Bueno et al. 2011).

In order to submit the animals in CT Group to the same “stress” as that of Group CG (moving the rats from their cages), the animals in CT Group were also moved every day, at the same time, to a transparent acrylic box, exactly the same as the CG Group but without exposure to cigarette smoke. After exposure to cigarette smoke or displacement, animals in both groups were returned to their conventional cages.

The animals were weighed weekly. The consumption of liquid and solid diet by the rats was also measured daily to calculate the average liquid and caloric intake, ruling out the possibility of malnutrition or dehydration during the experiment.

### 2.3 Euthanasia and Sample Collection

After 12 weeks, the animals were euthanized by anesthetic overdose (ketamine and xylazine, 0.20 mL/100 g IM), followed by laparotomy. Right kidneys were excised, weighed, and prepared for histological analysis.

### 2.4 Processing and Histomorphometric Analysis of the Kidney

After euthanasia, a laparotomy was performed and the right kidney of each rat was collected and weighed on an electronic analytical balance with a precision of 0.001 g, obtaining the wet weight of the kidney. The rat kidney tissue was fixed in 10% formalin for 48 h, stored in 70% alcohol, and embedded in paraffin for 4 μm thick histological sections. The histological sections were stained with hematoxylin and eosin for histomorphometric analysis in order to measure the glomerular diameter (μm), capsular area or Bowman's space (μm^2^), subcapsular area (μm^2^), and glomerular tuft area (μm^2^) (Junqueira and Brentani 2020; Ortiz et al. 2001). Using photomicrographs of histological sections of the kidneys, histological and morphometric analyses were carried out, with the histological sections being analyzed with the objective lens of the Binocular Biological light microscope—ECLIPSE E200 (Nikon), standardized at 10 X magnification (Carl Zeiss) for all histological sections in photomicrographs. Morphometry was performed using Image Lab software (version 5.2.1). All analyses were carried out by the same researcher through blind reading of the slides.

### 2.5 Obtaining Kidney Weight

The kidneys were wet weighed on an analytical balance. Kidney weight was corrected by the animal's weight on the day of euthanasia (PRenal/PRats × 100) (Awazu et al. 2003).

### 2.6 Statistical Analysis

The results were presented as means and standard deviations. For statistical analysis of the results, the GraphPad Prism 9.1 Program was used, in which Analysis of Variance (ANOVA) was applied at 5% significance, suitable for comparing groups by Tukey test.

## 3 Results and Discussion

Nicotine has a well‐known deleterious effect, such as the development of heart disease, cancer, or chronic obstructive pulmonary disease. The impact on kidney function has only recently been recognized and, as a result, there are not many studies available on its effect on the kidneys. According to epidemiological studies, smoking was the cause of the development of proteinuria, progression of diabetic nephropathy, and progression to end‐stage CKD (Speeckaert et al. 2013). Smoking is an important risk factor for the incidence of CKD in the healthy adult population in general (Noborisaka 2013). In this sense, the risk of incidence increases significantly and proportionally to the total number of pack/years and decreases when cessation occurs (Xia et al. 2017). However, despite being a relevant reduction, the risk in ex‐smokers remains higher than in the general population and may persist for more than 10 years after cessation as a result of smoking‐induced changes in the epigenetics of blood platelets (Xia et al. 2017). Furthermore, the renal toxic effects of smoking are more prevalent in elderly people, increasing the risk of incidence of CKD in this population (Noborisaka 2013). Exposure to cigarette smoke was, in the present study, the selected method to measure the effects of smoking on the renal morphology of rats—a relevant topic given the large number of smokers and the scarcity of studies associating renal morphological findings with the habit of smoking. The choice of six cigarettes per day was based on an “average” smoker, whose exposure to smoke would be sufficient for biological findings without exposing the animals to excessive toxicity from the harmful agents in smoke.

**FIGURE 1:**
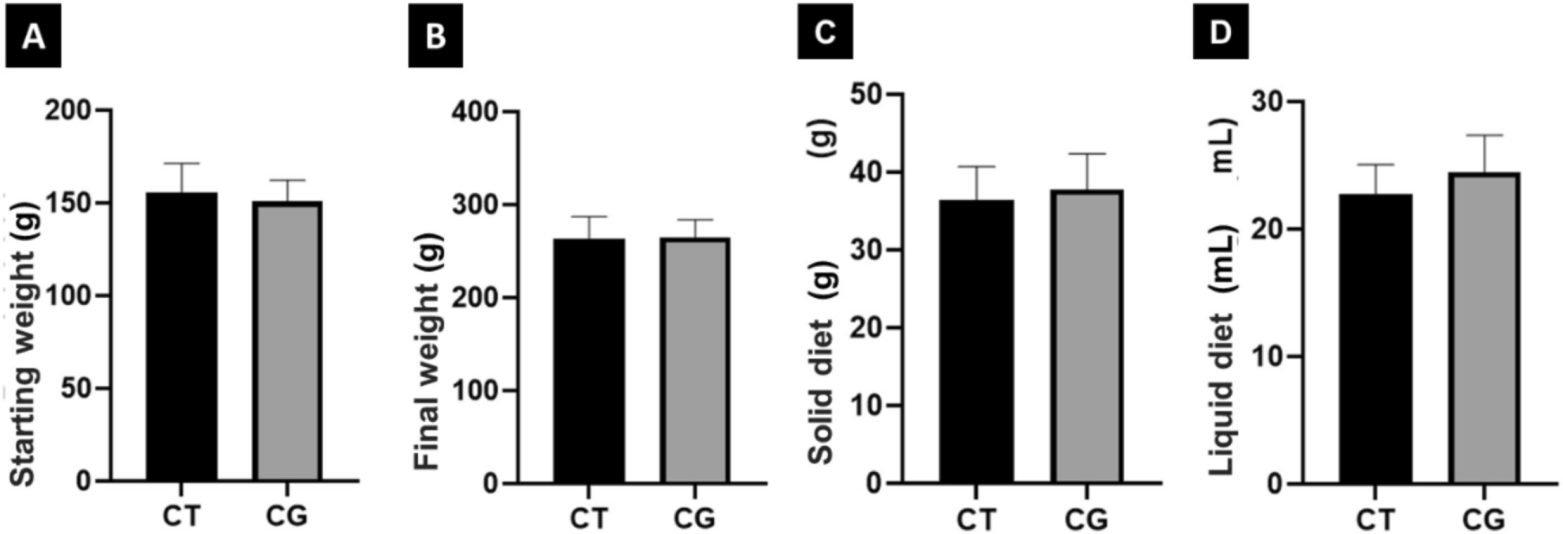
(A) Comparison of the initial weight (g) of the animals; (B) Final weight (g) of them; (C) Consumption of solid diet (feed) (g) per day; (D) Consumption of liquid diet (water) (mL) per day. There was no statistical difference in initial weight between the CT and CG Groups (*p* = 5580); the same for the comparison of the final weight between the two groups (*p* = 0.9359). There was no statistical difference between the CT and CG groups for food consumption (*p* = 5765) and water consumption (*p* = 0.2733).

Due to the fact that the present study exposed the animals to cigarette smoke, care was taken with their nutrition and hydration throughout the experiment. It was observed that the rats exposed to cigarette smoke (CG Group) and those in the CT gained weight throughout the experiment and showed no statistical differences when comparing the weights at the beginning and end of the experiment (Figure 1).

Experiments involving prolonged drug use require special attention regarding the nutritional status of the rats, as variations in the consumption of solid and liquid diets can cause changes in their biological responses during the experimental protocol. The consumption of water and food, defined as ideal to maintain the adequate nutritional status of rats and avoid malnutrition and dehydration, is, respectively, 15–80 mL of water and 25 g of feed per day (Soares et al. 2010). The data presented in Figure 1 demonstrated that exposure to cigarette smoke did not cause malnutrition or dehydration in the rats, as the animals gained weight throughout the experiment, so the findings of the present study are attributed exclusively to the harmful effects of cigarettes.

**FIGURE 2:**
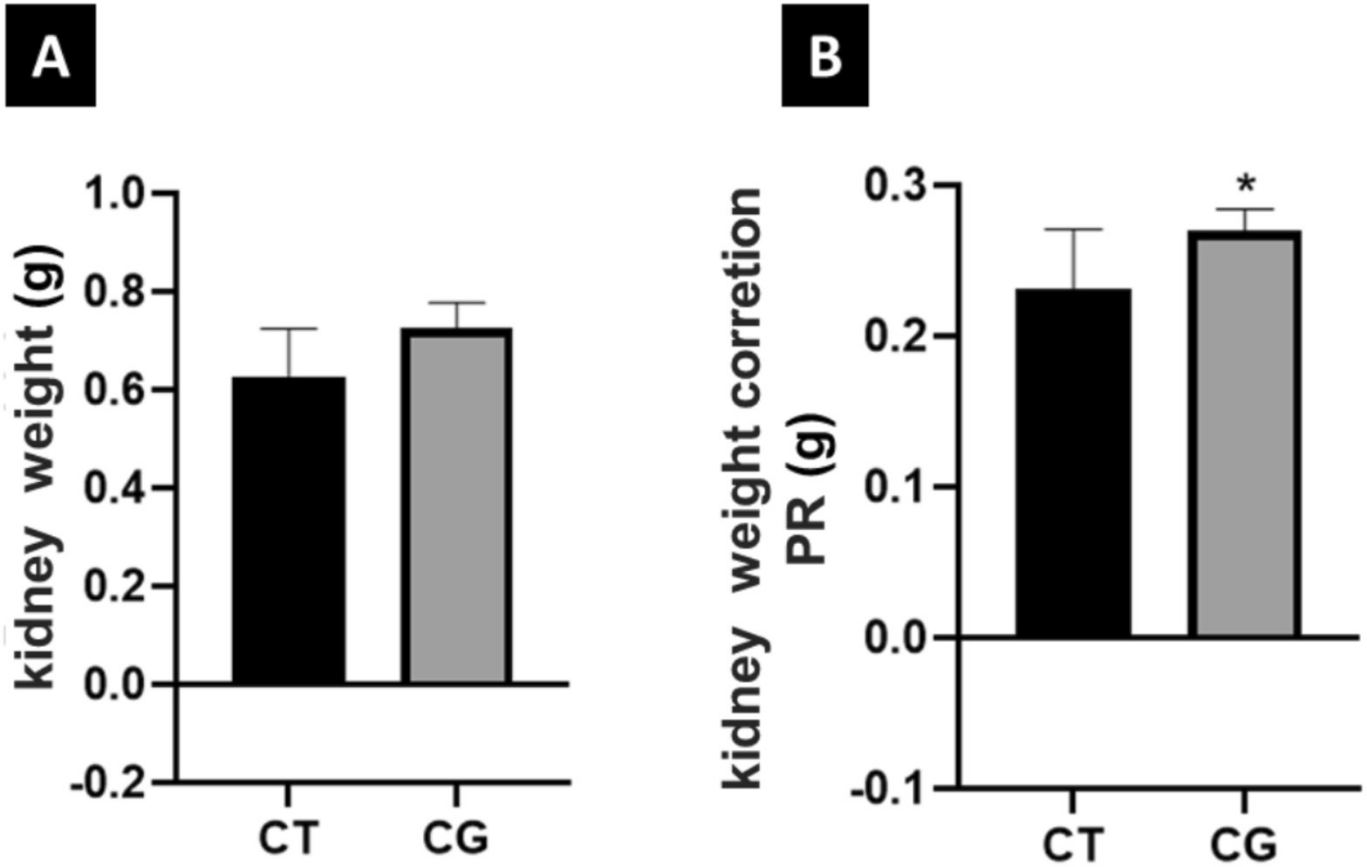
—Kidney weight (g) and CG and correction of kidney weight (g) of rats in CT Group. (A) There was no significant difference between the groups (*p* = 0.0548); (B)*statistically significant difference between CT and CG (*p* = 0.479) after correction.

The rat's kidneys from both groups were weighed on the day of euthanasia, and statistical analysis showed no difference in weight between animals from CT and CG groups. To correct the animals' kidney weight, the following calculation was performed: kidney weight/animal weight × 100. The data obtained from each group were compared and demonstrated that the rats in the CG Group (cigarette) presented higher values of corrected kidney weight than those in the CT (Figure 2).

Nicotine acts by deregulating essential biological processes, such as angiogenesis, apoptosis, and cell‐mediated immunity. Apoptosis is the process of programmed cell death and plays an important role in kidney growth and remodeling. As a high concentration of nicotine is observed in the blood and kidneys of smokers, the renal tubular cells are exposed to this substance. The epithelial cells of the proximal tubules are highly susceptible to apoptosis, and injury in this location favors the development of renal failure (Kim et al. 2016). In the present study, a detailed analysis of the vascular histology of the kidneys was not performed; however, the morphological findings that are presented below may establish relationships with changes in renal blood flow due to the experimental smoking to which the rats were submitted.

**FIGURE 3:**
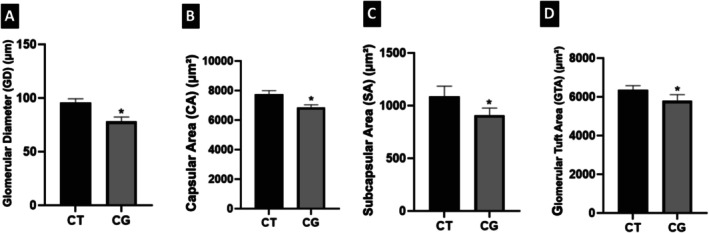
—Histomorphometry of glomerular diameter (GD) (μm), capsular area (CA) (μm^2^), subcapsular area (SA) (μm^2^) and glomerular tuft area (GTA) (μm^2^) of rats in CT and CG groups.*Statistically significant difference between CT and CG Groups in (A) and (B)—*p* < 0.0001; (C)—*p* = 0.0045; (D)—*p* = 0.0049.

**FIGURE 4:**
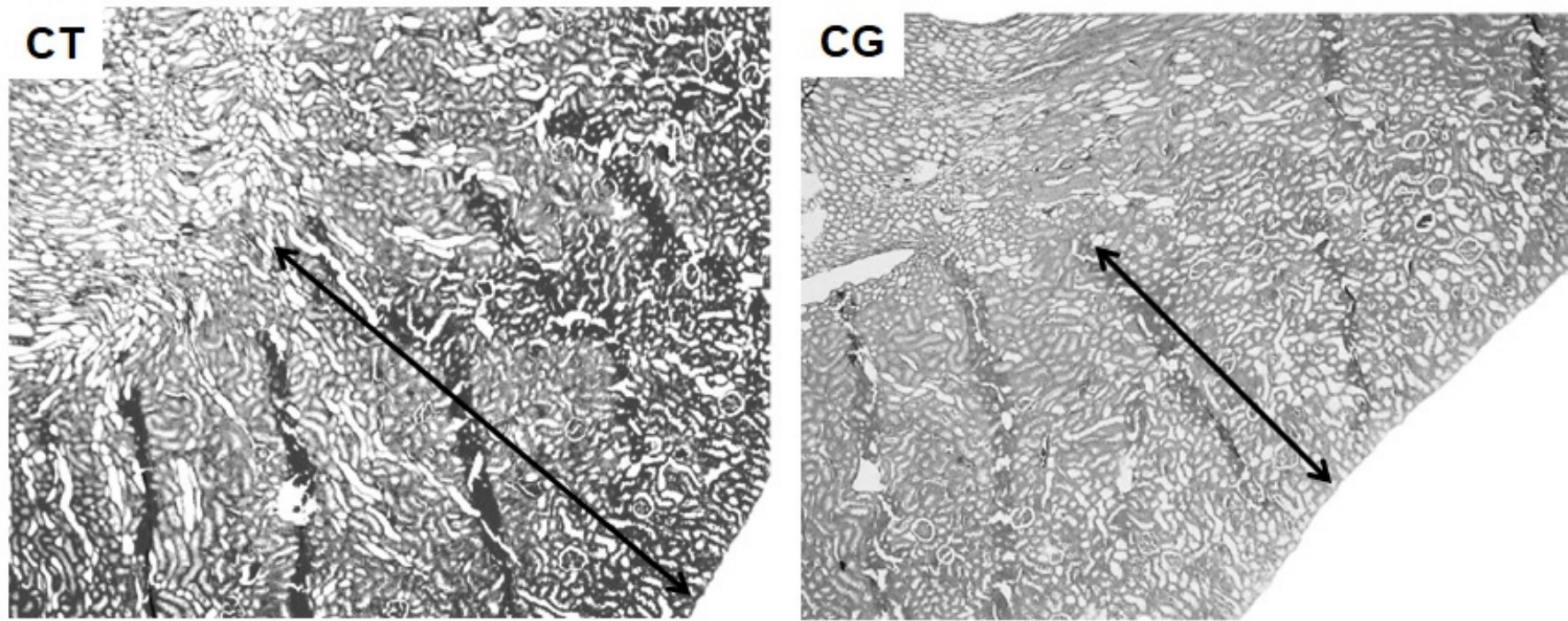
—Transverse photomicrograph of the right kidney of rats from CT and CG Groups demonstrating the cortical region (arrow) and medulla (no arrow) of the kidneys (HE 100 X).

**FIGURE 5:**
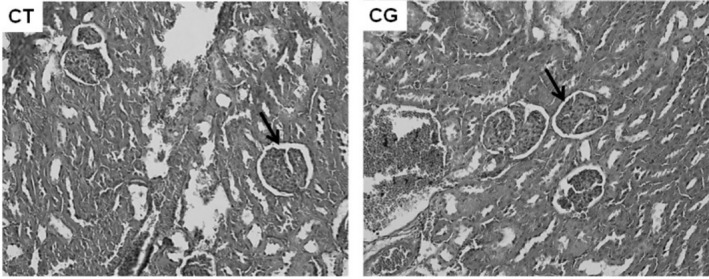
—Transverse photomicrograph of the right kidney of rats from CT and CG Groups demonstrating the renal glomeruli and the difference in the size of Bowman's space (arrow) (HE 100 X).

The animals in the CG Group presented measurements of glomerular diameter (μm), capsular area or Bowman's space (μm^2^), subcapsular area (μm^2^) and glomerular tuft area (μm^2^) smaller than those from the rats in the CT Group (Figure 3; Figures 4 and 5). Likewise, other researchers (Kaplan et al. 2020) also obtained smaller measurements of Bowman's space and glomerular tuft area in male rats in the CG when compared to male rats in the control group.

In this context, the photomicrograph in Figure 5 is in agreement with a study (Kaplan et al. 2020), in which male rats in the CG presented lower values for diameter and tubular lumen when compared to values in the control group, consistent with a picture of chronic tubular atrophy which suggests advanced glomerulotubular damage.

Tubular atrophy is a disease that predicts the progression of CKD and is frequently associated with interstitial fibrosis, being defined as the disappearance of individual tubular epithelial cells or entire tubules (Schelling 2016). This condition correlates detrimentally with serum creatinine, creatinine clearance, and the ability to concentrate and acidify urine, as well as with the clearance of inulin and para‐aminohippurate, the latter occurring when there is concomitant vascular disease (Schelling 2016). Furthermore, histopathological changes due to interstitial fibrosis and tubular atrophy are used as important prognostic and predictive markers for determining the degree of chronicity and evolution of kidney disease (Ginley et al. 2020).

Moreover, acute exposure to nicotine can decrease the growth of normal renal tubular epithelial cells, causing acute kidney injury due to increased expression of TP53, an important cell cycle regulatory gene (Chang and Singh 2018). On the other hand, long‐term exposure to nicotine can increase the survival and proliferation of these cells, due to the decrease in TP53 expression, which can lead to proliferative diseases, such as kidney cancer, and act as a potential initial event that leads to malignant transformation of these cells (Chang and Singh 2018; Huang et al. 2022).

Simultaneously, chronic nicotine exposure increased the extent of ischemia–reperfusion‐induced renal injury, as evidenced by morphological changes. Kidney injury is also due to increased production of reactive oxygen species (ROS) inducing oxidative stress. This smoking‐induced oxidative stress leads to endothelial dysfunction and vascular injury (Arany et al. 2011). Besides that, ROS act as mediators of COX‐2 expression, stimulating it in response to nicotine (Jaimes, Tian, et al. 2009). Also, it is suggested that nicotine‐induced oxidative stress causes epigenetic changes that contribute to the neoplastic transformation of human renal epithelial cells (Chang and Singh 2019).

Nicotine also increases the expression of cyclooxygenase‐2 (COX‐2) in mesangial cells and prostaglandins derived from COX‐2, as well as stimulates the proliferation of glomerular cells, the expression of fibronectin, and the production of extracellular matrix (Rangarajan et al. 2020; Jaimes, Tian, et al. 2009). This suggests that prostaglandins derived from COX‐2 may be important as mediators in the pathogenesis of kidney injury in response to smoking, accelerating its progression (Jaimes, Tian, et al. 2009). In this sense, prostaglandins in the kidney can have beneficial or harmful effects depending on their type and the pathological situation, with the imbalance caused by nicotine being a potential mechanism that can accelerate the progression of CKD (Rangarajan et al. 2020). Further, the effects of exposure to nicotine using the COX‐2 inhibitor NS‐398 can be attenuated, mainly in relation to fibronectin expression, glomerular injury, and proteinuria (Rangarajan et al. 2020).

Moreover, smoking patients who had clear cell carcinoma had more aggressive forms of renal cell carcinoma with worse medical comorbidities and worse survival prognoses when compared to non‐smoking patients (Kroeger et al. 2011). It was also found that smoking was associated with multifocal tumors, worse pT classification, and an increased rate of lymphatic and distant metastases. Expression of the tumor suppressor gene TP53 was detected more frequently in smokers than in non‐smokers or ex‐smokers and was considered an independent predictor of cancer‐specific survival in patients with non‐metastatic disease (Kroeger et al. 2011).

In the present study, the analyses carried out aimed to describe renal morphological changes upon exposure to cigarette smoke, and the presented findings suggest harmful effects not only of nicotine, but of all other components contained in cigarette smoke. The data obtained highlight the need for continued investigations regarding the molecular and functional aspects of the kidneys related to the harmful effects of cigarette smoking.

## 4 Conclusion

Exposure to cigarette, smoke altered the renal morphology of the animals, as evidenced by increased renal weight (although not statistically significant) and decreased values of glomerular diameter, capsular area (Bowman's space), subcapsular area, and glomerular tuft area. Therefore, these findings highlight the detrimental effects of cigarette smoke on kidney structure and, due to the large number of smokers, underscore the importance of public health initiatives focused on smoking prevention and cessation. Future research should incorporate functional assessments to further clarify the impact of cigarette smoke on renal physiology.

## Conflicts of Interest

The authors declare no conflicts of interest.

## Data Availability

The data that support the findings of this study are available on request from the corresponding author. The data are not publicly available due to privacy or ethical restrictions.

## References

[jat4872-bib-0001] Arany, I. , S. Grifoni , J. S. Clark , E. Csongradi , C. Maric , and L. A. Juncos . 2011. “Chronic Nicotine Exposure Exacerbates Acute Renal Ischemic Injury.” American Journal of Physiology. Renal Physiology 301, no. 1: F125–F133. 10.1152/ajprenal.00041.2011 21511693. 21511693 PMC3129886

[jat4872-bib-0002] Awazu, M. , S. Omori , K. Ishikura , M. Hida , and H. Fujita . 2003. “The Lack of Cyclin Kinase Inibitor P27^kip1^ Ameliorates Progression of Diabetic Nephropathy.” Journal of the American Society of Nephrology 14: 699–708. 12595506 10.1097/01.asn.0000051726.41601.c0

[jat4872-bib-0003] Bueno, A. C. , K. G. Ribeiro , T. M. Cruz , et al. 2011. “Efeitos da Fumaça de Cigarro Sobre a Osteogênese e a Resistência Mecânica Óssea em Ratos.” Revista de Ciências Farmacêuticas Básica e Aplicada 32, no. 1: 211–215.

[jat4872-bib-0005] César‐Neto, J. B. , B. B. Benatti , E. A. Sallum , M. Z. Casati , and F. H. Nociti Jr. 2006. “The Influence of Cigarette Smoke Inhalation and Its Cessation on the Tooth‐Supporting Alveolar Bone: A Histometric Study in Rats.” Journal of Periodontal Research 41, no. 2: 119–123. 10.1111/j.1600-0765.2005.00844.x 16499714

[jat4872-bib-0006] Chang, Y. U.‐W. E. I. , and K. A. M. A. L. E. S. H. W. A. R. P. Singh . 2018. “Duration‐Dependent Effects of Nicotine Exposure on Growth and AKT Activation in Human Kidney Epithelial Cells.” Molecular and Cellular Biochemistry 448, no. 1: 51–60. 29396723 10.1007/s11010-018-3312-1

[jat4872-bib-0007] Chang, Y. W. , and K. P. Singh . 2019. “Nicotine‐Induced Oxidative Stress Contributes to EMT and Stemness During Neoplastic Transformation Through Epigenetic Modifications in Human Kidney Epithelial Cells.” Toxicology and Applied Pharmacology 374: 65–76. 10.1016/j.taap.2019.04.023. 31047982

[jat4872-bib-0008] Elihimas Júnior, U. F. , H. C. Elihimas , V. M. Lemos , et al. 2014. “Tabagismo Como Fator de Risco Para a Doença Renal Crônica: Revisão Sistemática.” Jornal Brasileiro de Nefrologia 36, no. 4: 519–528. 10.5935/0101-2800.20140074. 25517282

[jat4872-bib-0009] Ginley, B. , K. Y., Jen , A., Rosenberg , et al. 2020. “Neural Network Segmentation of Interstitial Fibrosis, Tubular Atrophy, and Glomerulosclerosis in Renal Biopsies.” arXiv preprint arXiv. 2002.12868.

[jat4872-bib-0010] Gueutin, V. , G. Deray , and C. Isnard‐Bagnis . 2012. “Physiologie Rénale [Renal Physiology].” Bulletin du Cancer 99, no. 3: 237–249. 10.1684/bdc.2011.1482. 22157516

[jat4872-bib-0033] Guyton, A. C. , and J. E. Hall . 2020. Guyton and Hall Textbook of Medical Physiology. 14th ed. Elsevier.

[jat4872-bib-0012] Huang, Y. , Q. Wang , Y. Tang , et al. 2022. “Identification and Validation of a Cigarette Smoke‐Related Five‐Gene Signature as a Prognostic Biomarker in Kidney Renal Clear Cell Carcinoma.” Scientific Reports 12, no. 1: 2189. 10.1038/s41598-022-06352-y. 35140327 PMC8828851

[jat4872-bib-0013] Jaimes, E. A. , R.‐X. Tian , M. S. Joshi , and L. Raij . 2009. “Nicotine Augments Glomerular Injury in a Rat Model of Acute Nephritis.” American Journal of Nephrology 29, no. 4: 319–326. 18849602 10.1159/000163593 PMC7265426

[jat4872-bib-0014] Jaimes, E. A. , M. S. Zhou , M. Siddiqui , et al. 2021. “Nicotine, Smoking, Podocytes, and Diabetic Nephropathy.” American Journal of Physiology. Renal Physiology 320, no. 3: 442–453. 10.1152/ajprenal.00194.2020. PMC7988804 33459165

[jat4872-bib-0035] Jiang, S. , D. V. Quan , J. H. Sung , M‐Y. Lee , and H. Ha . 2019. “Cigarette Smoke Inhalation Aggravates Diabetic Kidney Injury in Rats.” Toxicology Research 8, no. 6: 964–971. 32704346 10.1039/c9tx00201d PMC7364853

[jat4872-bib-0034] Junqueira, L. C. , and R. R. Brentani . 1979. Basic Histology: Text & Atlas. 5th ed. Appleton & Lange.

[jat4872-bib-0015] Junqueira, L. C. , J. Carneiro , and P. Abrahamsohn . 2017. Histologia Básica: Texto e Atlas. 13th ed. Guanabara Koogan.

[jat4872-bib-0016] Kaplan, A. , E. Abidi , N. J. Habeichi , et al. 2020. “Gender‐Biased Kidney Damage in Mice Following Exposure to Tobacco Cigarette Smoke: More Protection in Premenopausal Females.” Physiological Reports 8, no. 2. 10.14814/phy2.14339. PMC6981307 31981316

[jat4872-bib-0018] Kim, C. S. , J. S. Choi , S. Y. Joo , et al. 2016. “Nicotine‐Induced Apoptosis in Human Renal Proximal Tubular Epithelial Cells.” PLoS ONE 11, no. 3: e0152591. 10.1371/journal.pone.0152591. 27028622 PMC4814027

[jat4872-bib-0019] Kroeger, N. , T. Klatte , F. D. Birkhäuser , et al. 2011. “Smoking Negatively Impacts Renal Cell Carcinoma Overall and Cancer‐Specific Survival.” Cancer 118, no. 7: 1795–1802. 10.1002/cncr.26453. 21997347

[jat4872-bib-0020] Kurtz, A. 2017. “Endocrine Functions of the Renal Interstitium.” Pflügers Archiv / European Journal of Physiology 469, no. 7‐8: 869–876. 10.1007/s00424-017-2008-9. 28624952

[jat4872-bib-0021] Lee, P. N. , and M. W. Fariss . 2017. “A Systematic Review of Possible Serious Adverse Health Effects of Nicotine Replacement Therapy.” Archives of Toxicology 91, no. 4: 1565–1594. 10.1007/s00204-016-1856-y. 27699443 PMC5364244

[jat4872-bib-0032] Moore, K. L. , A. F. Dalley , and A. M. R. Agur . 2024. Anatomia orientada para a clínica. 9th ed. Guanabara Koogan.

[jat4872-bib-0023] Noborisaka, Y. U. K. A. 2013. “Smoking and Chronic Kidney Disease in Healthy Populations.” Nephro‐Urology Monthly 5, no. 1: 655–667. 23577327 10.5812/numonthly.3527 PMC3614318

[jat4872-bib-0024] Orth, S. R. 2002. “Cigarette Smoking: An Important Renal Risk Factor ‐ Far Beyond Carcinogenesis.” Tobacco Induced Diseases 1, no. 2: 137–155. 10.1186/1617-9625-1-2-137. 19570254 PMC2671650

[jat4872-bib-0036] Ortiz, L. A. , A. Quan , A. Weinberg , and M. Baum . 2001. “Effect of Prenatal Dexamethasone on Rat Renal Development.” Kidney International 59, no. 5: 1663–1669. 11318936 10.1046/j.1523-1755.2001.0590051663.x PMC4127466

[jat4872-bib-0026] Rangarajan, S. , G. Rezonzew , P. Chumley , et al. 2020. “COX‐2‐Derived Prostaglandins as Mediators of the Deleterious Effects of Nicotine in Chronic Kidney Disease.” American Journal of Physiology. Renal Physiology 318, no. 2: F475–F485. 31841390 10.1152/ajprenal.00407.2019 PMC7052654

[jat4872-bib-0027] Schelling, J. R. 2016. “Tubular Atrophy in the Pathogenesis of Chronic Kidney Disease Progression.” Pediatric Nephrology 31, no. 5: 693–706. 10.1007/s00467-015-3169-4. 26208584 PMC4726480

[jat4872-bib-0028] Silverthorn, D. U. 2017. Fisiologia Humana: Uma Abordagem Integrada. 7th ed. Artmed.

[jat4872-bib-0029] Soares, E. A. , W. J. Fávaro , V. H. A. C. Quitete , C. A. Bertran , and J. A. Camilli . 2010. “Effects of Alcohol and Nicotine on the Mechanical Resistance of Bone and Bone Neoformation Around Hydroxyapatite Implants.” Journal of Bone and Mineral Metabolism (English ed. Print) 28: 101–107. 10.1007/s00774-009-0115-1 19669082

[jat4872-bib-0030] Speeckaert, M. M. , J. R. Delanghe , and R. C. Vanholder . 2013. “Chronic Nicotine Exposure and Acute Kidney Injury: New Concepts and Experimental Evidence.” Nephrology, Dialysis, Transplantation 28, no. 6: 1329–1331. 10.1093/ndt/gft019. 23449342

[jat4872-bib-0031] Xia, J. I. A. , J. Xia , L. Wang , et al. 2017. “Cigarette Smoking and Chronic Kidney Disease in the General Population: A Systematic Review and Meta‐Analysis of Prospective Cohort Studies.” Nephrology, Dialysis, Transplantation 32, no. 3: 475–487. 10.1093/ndt/gfw452 28339863

